# A bird distribution model for ring recovery data: where do the European robins go?

**DOI:** 10.1002/ece3.977

**Published:** 2014-02-14

**Authors:** Fränzi Korner-Nievergelt, Felix Liechti, Kasper Thorup

**Affiliations:** 1 Swiss Ornithological InstituteCH-6204, Sempach, Switzerland; 2 Natural History Museum of DenmarkDK-2100, Copenhagen, Denmark

**Keywords:** Bayesian analysis, large-scale distribution, leap-frog migration, mark reencounter data, migratory connectivity, ring recovery data, ring recovery model

## Abstract

For the study of migratory connectivity, birds have been individually marked by metal rings for more than 100 years. The resulting ring recovery data have been compiled in numerous bird migration atlases. However, estimation of what proportion of a particular population is migrating to which region is confounded by spatial heterogeneity in ring recovery probability. We present a product multinomial model that enables quantifying the continent-wide distribution of different bird populations during different seasons based on ring recovery data while accounting for spatial heterogeneity of ring recovery probability. We applied the model to an example data set of the European robin *Erithacus rubecula*. We assumed that ring recovery probability was equal between different groups of birds and that survival probability was constant. Simulated data indicate that violation of the assumption of constant survival did not affect our estimated bird distribution parameters but biased the estimates for recovery probability. Posterior predictive model checking indicated a good general model fit but also revealed lack of fit for a few groups of birds. This lack of fit may be due to between-group differences in the spatial distribution on smaller scales within regions. We found that 48% of the Scandinavian robins, but only 31% of the central European robins, wintered in northern Africa. The remaining parts of both populations wintered in southern and central Europe. Therefore, a substantial part of the Scandinavian population appears to leap over individuals from the central European population during migration. The model is applied to summary tables of numbers of ringed and recovered birds. This allows us to handle very large data sets as, for example, those presented in bird migration atlases.

## Introduction

The study of migratory connectivity is important to understand the ecology and population dynamics of species (Webster et al. 2002). Ecological conditions encountered during the nonbreeding period can affect breeding performance due to carry-over effects (Norris and Marra 2007; Schaub et al. 2011; Harrison et al. 2013). For conservation of bird species, it is therefore valuable to know where individual birds are found throughout the course of a year. In addition, knowledge of migratory connectivity is important to assess the risk of avian transmitted diseases such as avian flu (Liu et al. 2005).

There are many challenges to studying the migratory pathways of birds, particularly smaller species. An ideal technique to study bird migration would be a device that is so small that it could be carried by small migratory species without affecting their behavior, while allowing long-distance tracking with high precision (Robinson et al. 2010). However, such a device does not yet exist (Robinson et al. 2010). The existing tracking techniques are subject to a trade-off between weight and temporal and spatial precision of the measurements.

GPS devices measure location with high precision (a few meters, e.g., Hünerbein et al. 2000). A GPS can be combined with a satellite transmitter to follow the track of a bird in real time (e.g., Kjellén et al. 1997). However, the high weight of GPS and satellite telemetry devices (normally >10 g, Robinson et al. 2010) prohibits the use of these techniques for birds smaller than around 200 g body mass (usually an additional weight of <5% of the body mass is accepted, e.g., Naef-Daenzer et al. 2001). Even for larger species, researchers often only equip those individuals that are heavier than a threshold weight (e.g., Sauter et al. 2012). This produces a nonrandom sample of the population being studied. They are also very expensive, limiting the number that can be deployed.

Lightweight-tracking devices are data loggers such as light-based geolocators (e.g., Stutchbury et al. 2009; Bächler et al. 2010). The smallest geolocator now weighs around 0.6 g (Bridge et al. 2013) and can be applied to birds with a body weight of at least 10 g. However, the derivation of the location from light measurements is associated with large errors (up to several 100 km) depending on the season, the behavior of the bird and the shading the bird has experienced during its journey (Lisovski et al. 2012). Furthermore, as the birds have to be recaptured in order to retrieve the data, only those individuals that survive and return to the place of capture (normally the breeding site) can be tracked.

As an alternative to mounting a tracking device on the bird's body, analyzing the chemical composition of the feather can give information on the whereabouts of the bird during the time when the feathers grew. Commonly, isotopes (Hobson and Wassenaar 2008) or trace elements (Szép et al. 2003) are used for this purpose. However, the spatial resolution is normally very low and often it is only possible to determine whether or not two individuals used similar wintering areas.

In contrast, the most widely used method of individually marking birds, using metal rings, can be applied to nearly all bird species, and it provides precise location information if the bird is found and its ring is reported to a ringing scheme. Marking birds with metal rings has been performed by many professionals and amateur ornithologists for more than a century (Mortensen 1901). Since that time, more than 100 million birds have been ringed, and several million recovery records have been collected in international databases such as the Euring database (http://www.euring.org). Given the large sample sizes and wide geographic area of coverage, these data would seem to be ideal for studying migratory connectivity, defined as the proportions of birds from different breeding populations migrating to different wintering areas (Webster et al. 2002).

However, ringing data present challenges for analyzing migratory connectivity, because the probability of finding and reporting a ring varies geographically and over time, resulting in a nonrandom sample of the population being studied (Perdeck 1977).

Numerous bird migration atlases (Zink and Bairlein 1995; Brewer et al. 2000; Fransson and Pettersson 2001; Wernham et al. 2002; Bakken et al. 2003; Bønløkke et al. 2006; Spina and Volponi 2008; Csörgő et al. 2009; Hüppop and Hüppop 2009; Saurola et al. 2013) have mapped data that link ringing with recovery locations. These bird migration atlases show where each bird species can go, but they do not allow us to quantify migratory connectivity, because the probability of finding a ringed bird and reporting its ring to a ringing scheme varies tremendously among different regions (Perdeck 1977; Fiedler et al. 2004; Clark et al. 2009; Korner-Nievergelt et al. 2010a). None of the migration atlases we know of have made an attempt to formally take into account recovery probability in their recovery maps.

A few approaches have been proposed for estimating geographic variation in recovery probabilities, using simulation (Lokki and Saurola 2004), using covariates for recovery probability (Cowen this volume) or by comparing groups of birds for which equal recovery probability is assumed (Busse and Kania 1977; Jenni 1987; Kania and Busse 1987). The latter method has been used in recovery models that aimed to describe migratory connectivity (Bauthian et al. 2007; Thorup and Conn 2009; Korner-Nievergelt et al. 2012).

Here, we present a further alternative of a large-scale spatial ring recovery model. The aim is to describe a model formulation that can potentially be used to account for spatially and temporally heterogeneous ring recovery probability in ring recovery maps for future migration atlases. Therefore, the model is simple so that it can potentially be applied to a large number of data sets from various species.

We apply the model to example data from the European robin *Erithacus rubecula*. The European robin is a partial migrant. It breeds all over Europe. The Scandinavian population migrates southwards. In southern and central Europe, the wintering area overlaps with the breeding area. The southernmost wintering sites are in northern Africa. However, little is known about what proportion of each population migrates and how far the migrants go.

## Methods

### Data

**Figure 1:**
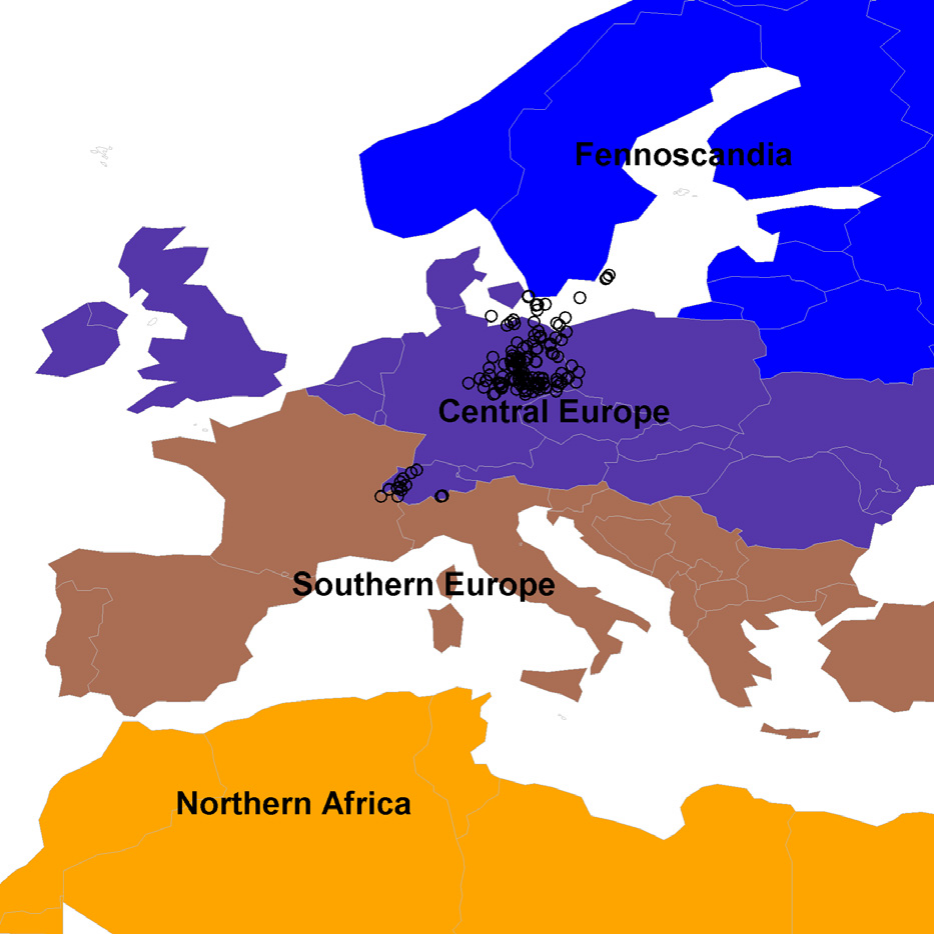
The four regions defined in this study. The circles identify the ringing locations.

We used ringing and recovery data for the European robin, which were kindly provided by the ringing stations Ottenby (http://www.sofnet.org/ottenbyfagelstation/start), Falsterbo (http://www.falsterbofagelstation.se), and Christiansö (Denmark) and the ringing schemes Hiddensee (Germany) and Switzerland (Fig. 1). Only data from birds ringed when fully grown were used. We used only reencounters of dead birds (i.e., recoveries) found more than 5 km from the place of ringing. This selection criterion ensures that the proportion of sedentary birds is not overestimated because ring recovery probability can be enhanced close to the ringing places (due to the activity of the ringers or the awareness of the people). Furthermore, we selected those recoveries for which the finding date was known with a precision of a least ±2 weeks. Recoveries from the month of ringing were discarded (i.e., only the first encounter per month was used). We only used data for birds ringed in 1964 or later, to ensure that all data sets were from a similar time period (Table 1).

**Table 1:** Description of the five data sets analyzed in this study with the total numbers of ringed European robins, the numbers of ringed birds in each month summed over the years, and the total number of ring recoveries.

Place of ringing	Ottenby (S)	Falsterbo (S)	Christiansö (DK)	Hiddensee (D)	Switzerland (CH)
Region	Fennoscandia	Fennoscandia	Fennoscandia	Central Europe	Central Europe
Years	1964–2011	1980–2011	1994–2002	1964–2011	2008–2011
Total numbers ringed	200,230	111,421	28,006	187,693	17,596
Numbers ringed in:					
January	0	0	0	222	8
February	0	0	0	173	2
March	2100	1650	135	7704	590
April	63,206	26,890	8961	40,328	2099
Mai	20,163	5227	1441	5672	6
June	142	52	37	2888	0
July	42	14	0	5690	28
August	520	2410	149	8258	436
September	54,348	46,072	10,255	65,214	5466
October	58,069	28,441	6789	48,729	8671
November	1633	665	239	2418	270
December	7	0	0	397	20
Total number of recoveries	366	185	45	540	16

We divided Europe into four regions: (A) Fennoscandia including Chistiansö, which is an island in the Baltic Sea south of Sweden; in this region, the European robin is a breeding or migratory bird, but it does not overwinter; (B) central Europe, which is both a breeding and a wintering area; (C) southern Europe; and (D) northern Africa (Fig. 1). Southern Europe (C) and northern Arica (D) can be considered as purely wintering areas for the European robins ringed in northern and central Europe. The robin also breeds in southern Europe and along the northern edge of northern Africa (Bauer et al. 2005). However, we assumed that we have no such birds in our data as all birds were ringed north of these regions.

We defined 12 release occasions according to the month of release. For each month, we summed data across years. Of the birds released in Fennoscandia, we can assume that they bred in Fennoscandia or in the northwestern part of Siberia. Among birds released in central Europe, only those released between May and July can be considered as breeding in central Europe. Birds ringed in central Europe during autumn, winter, and spring (August to April) have to be considered as a mixture of central European and Scandinavian birds as the Scandinavian birds migrate through or winter in central Europe during these months (Bauer et al. 2005).

### Model

We used a multistate model as described by Arnason (1972) and Schwarz (1993). We had four states representing the regions (*k* = A, B, C, D). The model described by Arnason (1972) and Schwarz (1993) estimates the transition probability that an individual moves to any possible state *k* at occasion *q* given it was in a specific state *k* at occasion 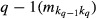. However, we formulated the model so that these transition parameters were independent of the state of an individual at occasion *q*-1 but instead depended on the group (or set) to which the bird belonged. Thus, in our model, these parameters represented the proportion of a set of birds (released in region *i* during month *j*) that were in region *k* during season *q* (*m*_*ijkq*_, see Table 2 for notation). We assigned the birds to 24 sets based on the ringing location (region A or B) and months (see Table 1 for number of ringed birds). The distributions of these sets of birds among the four regions were estimated for 8 seasons: winter (December to February), March, April, May, summer (June to August), September, October, and November. We assumed that the robins do not migrate during the three winter months and the three summer months, respectively. Note that for grouping the birds released, we use months here (indexed by *j*) but the distribution parameters are estimated per season (recovery occasion, which is sometimes a month and sometimes a group of months, indexed by *q*).

**Table 2:** Indices, parameters, and notation for data.

Data	
**R**_*ij*_	Vector of length K^*^Q + 1 containing the number of recoveries in each region (*k*) and season (*q*) of the set of birds released in region *i* during month *j*. The last number in the vector is the number of birds never recovered.
*N*_*ij*_	Number of ringed and released birds at region *i* during month *j*.
*I*	Total number of release regions (2)
*J*	Total number or release occasions, that is, months (12)
*K*	Total number of recovery regions (4)
*Q*	Total number of recovery occasions, that is, seasons (8)
Indices	
*i*	Release region, Fennoscandia and central Europe (A, B)
*j*	Release occasion, 12 months (1,…,12)
*k*	Recovery region, destination region (A, B, C, D)
*q*	Recovery seasons: 1,…8: winter, March, April, May, summer, September, October, November
*t*	Month (1,…., 12)
Model parameters	
**p**_*ij*_	Probability vector of length K^*^Q + 1
*p*_*ijkq*_	The first K^*^Q elements of **p**_*ij*_
*m*_*ijkq*_	The proportion of the set of birds *ij* (ringed in region *i* during month *j*) being in region *k* during season *q*
*F*_*jq*_	Probability that a bird alive during month *j* dies during a subsequent season *q*
*s*	Monthly survival probability
*r*_*kq*_	Recovery probability; probability that a ringed bird that has died in region *k* during season *q* is found and reported

We formulated one multinomial model for each set of birds ringed in the same region *i* and during the same month *j*.


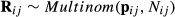


This yielded I*J = 2*12 = 24 models (one for each set of birds released), which together formed a product multinomial model.

**R**_*ij*_ is a vector of length K*Q + 1 containing the number of recoveries in each region *k* and season *q* for each set of birds (*ij*). The last element of the vector contained the number of birds that were never recovered.

The elements of the vector **p**_*ij*_ and the probabilities *p*_*ijkq*_ are the product of three probabilities: (1) the probability that a bird is in region *k* during season *q* given it was ringed in region *i* during month *j*,*m*_*ijkq*_, (2) the probability that a bird ringed and released during month *j* dies during a subsequent season *q*,*F*_*jq*_, and (3) the probability that a dead ringed bird is found and its ring reported to a ringing scheme, *r*_*kq*_.


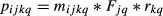


The probability that a ringed bird is never reported;


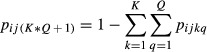


is added as the last element to **p**_*ij*_.

The probability that a bird ringed and released during month *j* dies during any subsequent season *q* (of any subsequent year), *F*_*jq*_, is obtained from the probabilities that a bird ringed and released during month *j* dies during a specific month *t* (in any subsequent year), 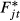. In our model, we ignore the year of recovery and instead only consider the month of recovery. If we assume that monthly survival *s* is constant over time and equal between the regions, three different cases have to be distinguished for the calculation of the probability 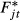. First, the month of ringing *j* is equal to the month of death *t*. In this case, the bird can survive 11 months and die in the 12th or it can survive 23 months and die in the 24th, etc. This gives a probability of 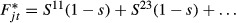. This is a geometric series that can be formulated as given in the first row of the formula below. When the bird dies later or earlier in the year than it was ringed, this probability has to be adjusted according to the difference in the number of months between the month of death and the month of ringing. These formulas are given in the second and third row below.


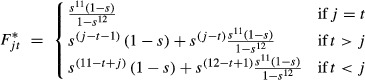


In order to obtain the probability that a bird ringed during month *j* dies during season *q* (instead of month *t*), we summed the corresponding 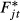, that is, 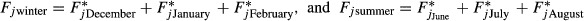. For the recovery seasons of one month duration (March, April, May, September, October, November) 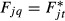.

The advantage of such a parameterization of survival in our model is that we do not need to add year of recovery as a fifth dimension (of length 48 in our case) in our data. This reduces computing time and enables data to be added from additional years without increasing the dimension of the data. One drawback is that we lose information about temporal variation in survival, but very large data sets would be needed to estimate that with precision anyway.

The model was fitted using Bayesian methods. A uniform distribution, Unif(0,1), was used as prior distribution for monthly survival probability *s*. We allowed for between-season variance in recovery probability, *r*_*kq*_, with partial pooling within each region. To do so, we modeled *r*_*kq*_ as a realization from a region-specific beta distribution: 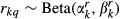 with Gamma priors for 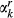 and 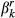 ∼ Gamma(0.01, 0.01) and 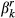 ∼ Gamma(0.01, 0.01).

For the bird distribution parameters *m*_*ijkq*_*,* we included the following constraints. Birds ringed during the three winter months (December–February) behaved similarly, thus *m*_*i1kq*_ = *m*_*i2kq*_ = *m*_*i12kq*_, and birds ringed during the months June and July were also assumed to behave similarly, that is, *m*_*i6kq*_ = *m*_*i7kq*_.

We used uniform prior distributions for 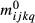 and transformed these so that the *m*_*ijkq*_ summed to 1 within one recovery season for each set of birds, 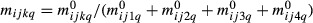. For most 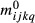, we used the Unif(0,1) prior distribution. However, because most of the European robins have left Sweden by September and are still in southern Europe in March (Fransson and Pettersson 2001), we used Unif(0, 0.01) as prior distributions for the proportions of birds in Fennoscandia between November and March. In this way, we constrained the proportions of birds wintering in Fennoscandia to be less than around 1%. Similarly, we constrained the proportions of birds spending the summer in northern Africa and southern Europe during summer to be less than around 1% for all sets of birds.

The model was fitted in JAGS using the package R2jags (Su and Yajima 2012). Two Markov chains of length 20,000 were simulated. Burnin was set to 5000, and the chains were thinned by two. Convergence was assessed visually and based on the Brooks–Gelman–Rubin statistics (Brooks and Gelman 1998). The JAGS code for the model is provided in the Data S1.

In our model, the bird distribution parameters are identifiable if there are at least as many sets of released birds (*ij*) as there are recovery regions and if the different sets of birds differ somewhat in their distribution among the regions (Korner-Nievergelt et al. 2010b). Here, we have 24 sets of birds and four regions. Thus, all bird distribution parameters should theoretically be estimable. However, if the data are too sparse or the spatial distribution of the different sets of birds is too similar, estimability of some parameters can be poor (Schaub 2009). Fitting the model to simulated data sets of different sample sizes indicated that for our study, some of the parameters may be little informed by the data (Data S2). Therefore, we compared the posterior distribution of each parameter with its prior distribution to assess how strongly the parameters were informed by the data. For this, we calculated the percentage by which the posterior distribution overlapped with the prior distribution (Garrett and Zeger 2000; Gimenez et al. 2009). Garrett and Zeger (2000) suggested that a parameter estimate can be considered well informed by the data when the overlap between the posterior and a uniform prior is less than 35%. For nonuniform priors, as used here for the parameters *m*_*ijkq*_, the threshold may be higher but no general guideline is available (Gimenez et al. 2009). Therefore, we used the overlap between the posterior and the prior distribution as a relative measure: The smaller the overlap, the more data-informed the parameter.

In order to assess model fit, we used predictive model checking (Gelman et al. 1996). To do so, we simulated recovery data based on the model while taking into account the uncertainty in the parameter estimates. We then graphically compared these predictive distributions with the corresponding numbers of observed recoveries.

## Results

Estimated monthly survival probability, *s*, was 0.89 (95% credible interval (CrI): 0.87–0.91, prior overlap: 6%). Recovery probabilities, *r*_*kq*_, were lowest in northern Africa and highest in central Europe (Table 3). During summer, the recovery probabilities were lower than during the migration periods. The mean overlap of the prior with the posterior distribution of *r*_*kq*_ was 1% (range 0.1–5%).

**Table 3:** Estimated probabilities that a robin that dies in each region in a particular season are found and its ring reported to a ringing scheme (recovery probability *r*_*kq*_). Estimates are means of the posterior distributions, and the numbers in brackets are standard deviations from the posterior distributions, that is, standard errors of the estimates.

Region	Winter	March	April	May	Summer	September	October	November
Fenno-scandia	0.0041^1^ (0.0029)	0.0073^1^ (0.0064)	0.0064 (0.0014)	0.0041 (0.0009)	0.0005 (0.0001)	0.0016 (0.0005)	0.0017 (0.0005)	0.0175^1^ (0.0093)
Central Europe	0.0021 (0.0007)	0.0041 (0.0013)	0.0134 (0.0027)	0.0035 (0.0010)	0.0004 (0.0001)	0.0031 (0.0010)	0.0056 (0.0012)	0.0025 (0.0007)
Southern Europe	0.0033 (0.0005)	0.0024 (0.0006)	0.0018 (0.0006)	0.0005 (0.0002)	0.0031 (0.0021)	0.0013 (0.0006)	0.0061 (0.0014)	0.0056 (0.0010)
Northern Africa	0.0010 (0.0002)	0.0009 (0.0003)	0.0001 (0.0001)	0.0001 (0.0001)	0.0041^1^ (0.0027)	0.0001 (0.0001)	0.0018 (0.0005)	0.0017 (0.0004)

1 Estimates are most likely influenced by our choice of the prior distribution of the proportion of birds being in the region during that season (*m*_*ijkq*_), which was Unif(0,0.01).

**Figure 2:**
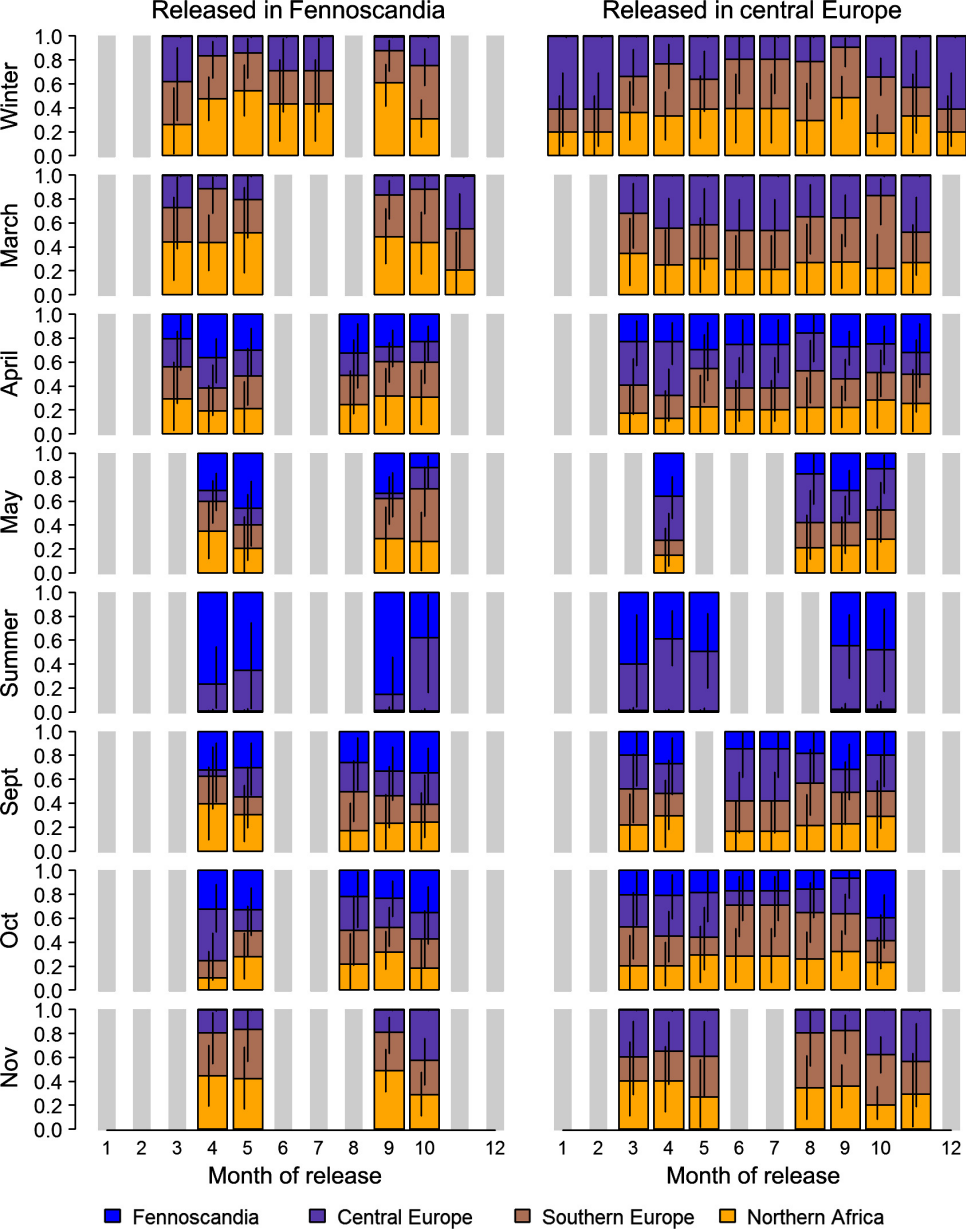
Estimated distribution during eight seasons (rows) of birds ringed in Fennoscandia and central Europe. The month of ringing (release) is given on the *x*-axis. The colors in the bars give the distribution of the birds among the four regions. Gray bars indicate distribution estimates for which the median overlap between the prior and the posterior distributions was higher than 95%. For these groups, the distribution estimates are not given. The vertical lines give the 95% credible intervals of the summed proportions as given in the figure. The 95% credible intervals for each single estimated proportion are given in the Data S3. For sample sizes, see Table 1.

**Figure 3:**
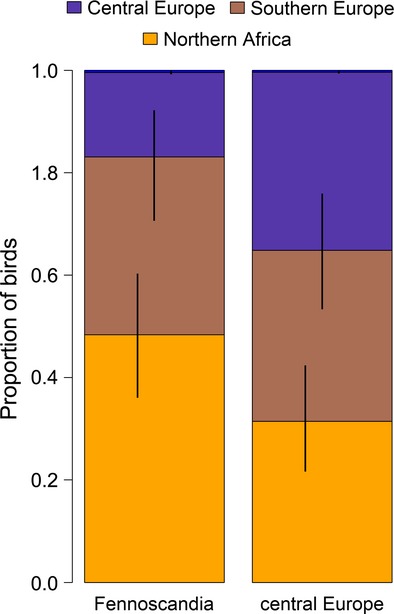
Mean of the estimated winter distribution (December–February) of birds ringed in Fennoscandia during all months with a sufficient high number of ringed birds (April, May, September, and October) and central Europe during the months May–August. The colors in the bars give the distribution of the birds among the three regions. The vertical lines give the 95% credible intervals.

Overlap between prior and posterior distributions for the bird distribution parameters *m*_*ijkq*_ ranged from 27 to 100% (mean 85%). The estimates were well data-informed for Scandinavian birds ringed during April, May, September, and October and for the central European birds ringed between March and October (Fig. 2, Data S3). Estimated proportions of birds spending the winter in northern Africa were higher for birds ringed in September than for birds ringed in October (Fig. 2). The average proportion of Scandinavian birds wintering in northern Africa is 48% (95% CrI: 36–60%, including only estimates that are well informed by the data, i.e., for birds ringed during April, May, September, and October Fig. 3). Of the birds ringed in central Europe during the breeding season (May to August), the average proportion that winters in northern Africa is significantly lower, that is, 31% (22–42%).

Overall, the model seems to fit the data well as assessed by posterior predictive model checking (Data S4). Two discrepancies between the model and the data deserve attention. For Scandinavian birds ringed in October, the model predicts a higher than observed number of recoveries in Fennoscandia during summer. We also find more recoveries during winter in central Europe of birds ringed in central Europe during December than predicted by the model.

## Discussion

The key assumption of the model presented here is that the (region (*k*)-and season (*q*)-specific) recovery probability does not depend on the region (*i*) and month (*j*) of ringing, that is, the different sets of birds (*ij*) are assumed to experience the same recovery probabilities given they are in the same region. This assumption could be violated if recovery probability is spatially heterogeneous within a region and the different sets of birds use different subregions within a region. For robins ringed in Sweden and at the southern Baltic coast, it has been stated that early migrants winter more westerly within the Mediterranean than late migrants (Högstedt and Persson 1971; Pettersson et al. 1990; Remisiewicz 2002). Furthermore, ring recovery probability decreases toward the east in the Mediterranean (Korner-Nievergelt et al. 2012). Therefore, robins ringed in September (early migrants) will winter more to the west and experience a higher recovery probability than robins ringed in October (late migrants). However, our model assumes the same recovery probability for both sets of birds as we have not divided the Mediterranean into an eastern and western region (Fig. 1). As a consequence, our model may underestimate the proportions of birds ringed in October that winter in southern Europe and northern Africa.

This implies that the difference in the proportion of birds wintering in northern Africa between the birds ringed in October and those ringed in September may appear because the later migrants winter more to the east than the earlier migrants (rather than because earlier migrants winter farther south). It would be valuable to distinguish the eastern and western Mediterranean as separate regions in the model. However, when we fitted such a model, most of the parameter estimates were not or very weakly informed by the data. Therefore, more data would be needed to fit such a model.

Another assumption made by the model is constant survival. For two migratory songbirds of similar size to the robin, the black-throated blue warbler *Dendroica caerulescens* and the barn swallow *Hirundo rustica*, it has been shown that survival is reduced in the first few weeks after fledging, but later it is similar to that of adults (Sillett & Holmes 2002; Grüebler and Naef-Daenzer 2010). We excluded birds ringed during or before the fledgling period by selecting only birds ringed when fully grown to reduce age dependency of survival in the data. However, our estimated monthly survival probability of 0.89 translates into an annual survival probability of 0.25. This estimate seems to be lower than or at the lower edge of annual survival estimates for the European robin given by other authors based on ringing and recovery data— Lack (1964): first year (including fledging period) 0.34, adults 0.38, and migrants with unknown age 0.23; Fransson and Pettersson (2001): first year 0.21 and adults 0.35; and Siriwardena et al. (1998): first year 0.40 and adults 0.42. Underestimation in survival probability can be caused by unaccounted heterogeneity in either survival or recovery probability (Carothers 1973; Gilbert 1973; Fletcher et al. 2012). Such heterogeneity could be nonconstant survival probability over the year. For example, in the black-throated blue warbler, survival was reduced during the migration periods compared with the rest of the year (Sillett & Holmes 2002).

In order to assess the effect of such nonconstant survival in the data on our model estimates, we simulated data based on the assumption of lower survival probability during the migration period than during winter and summer. Fitting the model to these data showed that nonconstant survival primarily affects the estimates for recovery probability, but has no effect on bias or precision of the distribution parameter estimates (Data S5). These simulations also show that the model in its present form is primarily designed for estimating bird distributions rather than analyzing survival.

A further point that deserves discussion is that it is not possible to identify the sex from plumage in the European robin. As a consequence, we cannot include sex-specific migration in our model. However, it is well known that a higher proportion of the females are migratory compared with males. For example, at a Belgian study site, 100% of the females were migratory, whereas only 0–70% of the males were migratory (Adriaensen and Dhont 1990). Unaccounted heterogeneity in some parameters can sometimes cause bias in the parameter estimates in mark–recapture models (Fletcher et al. 2012). Therefore, we simulated data for males and females separately assuming different migration patterns for females and males. We then pooled the data (i.e., ignoring sex as in the real data) and analyzed the data with our model. The bias of the distribution parameters *m*_*ijkq*_ was, for the settings used in our simulation study, negligible (−0.003, if the mean of the sex-specific distribution parameters was assumed to be the true value), and the precision of the estimates was similar to data sets of equal sample sizes without distributional differences between the sexes (Data S6). This means that the estimates corresponded, for our example, to the proportions of all birds (ignoring sex) in the different regions during the different seasons. It remains to be tested whether it is possible to account for such heterogeneity by extending the model with a mixture model as proposed by Pledger (2000).

While overall the model fitted the data well, the predictive model checking revealed some heterogeneities not accounted for in the model (Data S4). For example, predicted numbers of reencounters in Fennoscandia during the summer were too high for birds ringed in Scandinavia in October. This may be because, during autumn, populations from northeastern Fennoscandia are migrating through Europe and, thus, are ringed in Scandinavia and central Europe. These birds breed at places with much lower recovery probabilities than elsewhere in Fennoscandia. As a consequence, birds ringed during migration have a lower recovery probability during summer within Fennoscandia than birds ringed during the nonmigration period. As the model assumes that recovery probability does not depend on when the bird was ringed, we observe fewer recoveries than expected from those birds ringed during migration. That we do not see this discrepancy for Scandinavian birds ringed during spring migration may be because these northeastern breeding birds take more easterly routes in the spring. Such a loop migration for the northeastern breeding birds has been suggested by Pettersson et al. (1990) who analyzed morphological measurements of robins in southern Sweden and at different places in the wintering area.

Literature that looks at the proportions of birds from different populations that stay in different regions over the year is sparse. Schifferli (1961) wrote that only 5–10% of the robins breeding in Switzerland stay there over winter. Among 46 individual offspring of a population from southern Germany, 10 (22%) did not show migratory restlessness (Biebach 1983). Our model estimated this proportion as 36% (12–63%) for those birds ringed during May and 19% (0.8–48%) for those birds ringed during June and July. Our model estimates are slightly higher than those in the literature. In Belgium, all females left the study plots (of 10–70 ha size) in winter, whereas of the males only 70% left the woodland plots and 0% the park and garden plots. It is not known what proportion of these individuals left central Europe. However, the study shows that, especially in urban areas, robins may stay within less than 5 km of the breeding place to spend the winter. Because we discarded recoveries within less than 5 km distance from the place of ringing, our estimates of sedentary birds may be too low. Despite this underestimation, we have a higher proportion of sedentary birds than earlier studies. This difference may be due to a long-term change in migration behavior or simply due to uncertainty in all of these estimates as the number of ringed birds during the breeding season is low.

The proportions of birds wintering in northern Africa are consistently higher for those birds ringed in Scandinavia compared with those ringed in central Europe (Fig. 3). This means that many Scandinavian individuals overtake central European ones during autumn migration. This may indicate a leap-frog migration at least in parts of the population (Salomonsen 1955).

The motivation for this study was to develop a method to correct the ring recovery maps given in bird migration atlases for spatially heterogeneous ring recovery probabilities. The model we present here is an extension of previous bird distribution models used by Bauthian et al. 2007, Thorup and Conn 2009, and Korner-Nievergelt et al. 2012. We extended these models by including a seasonal dependence of the bird distribution without including a year dependence of any model parameter. The independence from year makes our model suitable for analyzing data sets that have been collected over a long time period (>50 years) with few observations per year.

The model enables quantification of the temporal and spatial distribution of bird populations as well as migratory connectivity. Based on the experience from this study, we recommend including as many individuals as possible in the data, including data from different species. As the assumption that recovery probabilities are equal between different species may not be realistic, species-specific recovery probabilities may need to be estimated with partial pooling between the species. In addition, other covariates for recovery probability, for example human density (Korner-Nievergelt et al. 2010a), may be added to the model. Finally, the regions should be chosen small enough so that it can be assumed that ring recovery probability is homogeneous within each region.

The application of this model to combined data of a large number of species with a high number of regions requires that the computation is fast. The models presented here needed around three minutes to be fitted to the present data set on an intel COREi5vPro notebook. This is fast enough to be confident that fitting a similar model to data of more regions and more sets of birds released will be feasible.

Finally, we would like to note that the Bayesian framework allows the inclusion of information from other data sources, such as modern tracking methods, either as prior information or as an integrated model, similar to the integrated population models (Abadi et al. 2010). Data sets from different sources often differ in quality, for example ring recovery data contain limited information about many individuals, primarily after death, whereas tracking data contain more information of only a few individuals that survived until the data could be downloaded. The combination of such different data sources has the potential to give a more comprehensive insight into the migration behavior of the species than two separate analyses. Our study contributes to the development of formal methods for the combination of different data sources that has been recognized as being important for the study of migratory connectivity (Norris et al. 2006; Baillie et al. 2009; Fiedler 2009).

## Supporting Information

Data S1 R and JAGS code for fitting the distribution model to ring reencounter data.

Data S2 The aim of this simulation study was to assess the sample size required to estimate the proportion of birds of the different sets of birds *(ij)* being in the different regions *k* during each season *q, mijkq*.

**Data S3.** This document contains two figures that show the 95% credible intervals of all proportions presented in the barplot of Fig. 2 of the main text. **Fig. S3.** (A) Estimated proportions of birds ringed in Fennoscandia during different months (indicated on the left outer axis) being in the four different regions (indicated on the bottom) during 8 different seasons (x-axis). (B) Legend as in Fig. S3a but for birds released in central Europe.

**Data S4.** Diagnostic figures from posterior predictive model checking for two models: (1) model assuming constant recovery probability over time (not presented in the main text), (2) model assuming season-dependent recovery probability (as presented in the main text). **Fig. S1.** Mean and 95% interval of the posterior predictive distributions of the number of recoveries [log ()] centered around the observed number of recoveries in each region for each season (bottom axis) for birds ringed in Fennoscandia during a specific month (left outer axis). **Fig. S2.** Legend as in Fig. S1 but for birds ringed in central Europe.

Data S5 The aim of this simulation study was to assess the influence of nonconstant survival on the estimates of the model parameter, as our model assumes constant survival probability.

Data S6 The aim of this supplementary material is to assess the effect of sex-specific migration on the model estimates when sex is not identified.
