# Supplementary material for: A bird distribution model for ring recovery data: where do the European robins go?
Source: Ecol Evol. 2014 Feb 14;4(6):720–31. doi: 10.1002/ece3.977 (PMC3967898; doi:10.1002/ece3.977)
Supplement: Data S1 — R and JAGS code for fitting the distribution model to ring reencounter data. [file ece30004-0720-sd1.docx]

**Data S1**

Korner-Nievergelt, Liechti, Thorup (2014): A bird distribution model for ring recovery data: Where do the European robins go? Ecology & Evolution

**R and JAGS-code for fitting the distribution model to ring reencounter data**

The data his provided in the “datax”-object that is a list with the following elements:

> str(datax)

List of 8

$ recmatlong: num [1:24, 1:33] 0 0 0 0 0 0 0 0 1 1 ...

$ nreleased : num [1:24] 5 5 3885 99057 26831 ...

$ npop : num 2

$ nrelocc : num 12

$ ndest : num 4

$ nrecocc : num 8

$ behgroup : num [1:12] 1 1 2 3 4 5 5 6 7 8 ...

$ nbehgroup : num 9

The element “recmatlong” corresponds to **R***_ij_* (see Table 2 in the manuscript), “nreleased” is a vector with the number of released birds in each set of bird, *N_ij_* (note that this vector cannot contain zeros, you can insert a small number, e.g. 3), “npop” is *I*, “nrelocc” is *J*, “ndest” is *K*, “nrecocc” is *Q*, “behgroup” is a vector that indicates the groups of birds released at different months that are assumed to behave similarly with respect to migration, and “nbehgroup” is the number of groups of birds that are assumed to behave similarly. Note, that the index “l” corresponds to the index “q” in the manuscript all other indices are used as in Table 2.

# define directories

bugsworkingdir <- getwd()

# load libraries

library(R2jags)

#-------------------------------------------------------------------------------

# Model definition

sink(file.path(bugsworkingdir, "distributionmodelrtime.txt"))

cat("

model{

### Likelihood

for(i in 1:(npop*nrelocc)){

recmatlong[i,1:33]~ dmulti(probmatlong[i,1:33], nreleased[i])

ynewlong[i,1:33]~ dmulti(probmatlong[i,1:33], nreleased[i])

}

# survival matrix

A <- pow(s,11)*(1-s)/(1-pow(s,12))

for(j in 1:nrelocc){

# diagonal

survivalmatcomp[j, j] <- A

}

# upper diagonal

for(j in 1:(nrelocc-1)){

for(lc in (j+1):nrelocc){

survivalmatcomp[j, lc] <- pow(s,(lc-j-1))*(1-s) + pow(s,(lc-j))*A

}

}

# lower diagonal

for(j in 2:nrelocc){

for(lc in 1:(j-1)){

survivalmatcomp[j, lc] <- pow(s,(11-j+lc))*(1-s) + pow(s,(12-j+lc))*A

}

}

# merge recovery seasons (winter and summer)

for(j in 1:nrelocc){

survivalmat[j,1] <- survivalmatcomp[j,1] + survivalmatcomp[j,2] + survivalmatcomp[j,12]

for(l in 2:4){

survivalmat[j,l] <- survivalmatcomp[j,l+1]

}

survivalmat[j,5] <- sum(survivalmatcomp[j,6:8])

for(l in 6:8){

survivalmat[j,l] <- survivalmatcomp[j,l+3]

}

}

# constraint for distributions

for(i in 1:npop){

for(j in 1:nrelocc){

# proportion of birds in A

m0[i,j,1,1] <- m0a[i,behgroup[j],1,1] # winter

m0[i,j,1,2] <- m0a[i,behgroup[j],1,2] # march

m0[i,j,1,3] <- m0a[i,behgroup[j],1,3] # april

m0[i,j,1,4] <- m0a[i,behgroup[j],1,4] # mai

m0[i,j,1,5] <- m0a[i,behgroup[j],1,5] # juni-aug

m0[i,j,1,6] <- m0a[i,behgroup[j],1,6] # sept

m0[i,j,1,7] <- m0a[i,behgroup[j],1,7] # oct

m0[i,j,1,8] <- m0a[i,behgroup[j],1,8] # nov

for(l in 1:nrecocc){

m0[i,j,2,l] <- m0a[i,behgroup[j],2,l] # proportion of birds in B

}

for(k in 3:ndest){ # in C and D

m0[i,j,k,1] <- m0a[i,behgroup[j],k,1] # winter

m0[i,j,k,2] <- m0a[i,behgroup[j],k,2] # march

m0[i,j,k,3] <- m0a[i,behgroup[j],k,3] # april

m0[i,j,k,4] <- m0a[i,behgroup[j],k,4] # mai

m0[i,j,k,5] <- m0a[i,behgroup[j],k,5] # juni-aug

m0[i,j,k,6] <- m0a[i,behgroup[j],k,6] # sept

m0[i,j,k,7] <- m0a[i,behgroup[j],k,7] # oct

m0[i,j,k,8] <- m0a[i,behgroup[j],k,8] # nov

} #k

} #j

} #i

# proportions are equal for those birds ringed during the winter and summer months

for(i in 1:npop){

for(j in 1:nbehgroup){

# proportion of birds in A

m0a[i,j,1,1] ~ dunif(0,0.01) # winter

m0a[i,j,1,2] ~ dunif(0,0.01) # march

m0a[i,j,1,3] ~ dunif(0,1) # april

m0a[i,j,1,4] ~ dunif(0,1) # mai

m0a[i,j,1,5] ~ dunif(0,1) # juni-aug

m0a[i,j,1,6] ~ dunif(0,1) # sept

m0a[i,j,1,7] ~ dunif(0,1) # oct

m0a[i,j,1,8] ~ dunif(0,0.01) # nov

# proportions of birds in B

m0a[i,j,2,1] ~ dunif(0,1)

m0a[i,j,2,2] ~ dunif(0,1)

m0a[i,j,2,3] ~ dunif(0,1)

m0a[i,j,2,4] ~ dunif(0,1)

m0a[i,j,2,5] ~ dunif(0,1)

m0a[i,j,2,6] ~ dunif(0,1)

m0a[i,j,2,7] ~ dunif(0,1)

m0a[i,j,2,8] ~ dunif(0,1)

# proportions of birds in C

m0a[i,j,3,1] ~ dunif(0,1) # winter

m0a[i,j,3,2] ~ dunif(0,1) # march

m0a[i,j,3,3] ~ dunif(0,1) # april

m0a[i,j,3,4] ~ dunif(0,1) # mai

m0a[i,j,3,5] ~ dunif(0, 0.01) # juni-aug

m0a[i,j,3,6] ~ dunif(0,1) # sept

m0a[i,j,3,7] ~ dunif(0,1) # oct

m0a[i,j,3,8] ~ dunif(0,1) # nov

# proportions of birds in D

m0a[i,j,4,1] ~ dunif(0,1) # winter

m0a[i,j,4,2] ~ dunif(0,1) # march

m0a[i,j,4,3] ~ dunif(0,1) # april

m0a[i,j,4,4] ~ dunif(0,1) # mai

m0a[i,j,4,5] ~ dunif(0, 0.01) # juni-aug

m0a[i,j,4,6] ~ dunif(0,1) # sept

m0a[i,j,4,7] ~ dunif(0,1) # oct

m0a[i,j,4,8] ~ dunif(0,1) # nov

} #j

} #i

# sum of m over destination areas = 1

for(i in 1:npop){

for(j in 1:nrelocc){

for(l in 1:nrecocc){

summ[i,j,l] <- sum(m0[i,j,1:ndest,l])

for(k in 1:ndest){

m[i,j,k,l] <- m0[i,j,k,l]/summ[i,j,l]

}

}

}

}

# fill up probability matrix

for(i in 1:npop){

for(j in 1:nrelocc){

for(k in 1:ndest){

for(l in 1:nrecocc){

probmat[i,j,k,l] <- survivalmat[j,l] * r[k,l] * m[i,j,k,l]

}

}

}

}

# rearrange probabilities into long format

for(i in 1:npop){

for(j in 1:nrelocc){

for(k in 1:ndest){

probmatlong[(i-1)*nrelocc+j, ((k-1)*nrecocc+1):(k*nrecocc)] <- probmat[i,j,k,1:nrecocc]

}

probmatlong[(i-1)*nrelocc+j, nrecocc*ndest+1] <- 1-sum(probmatlong[(i-1)*nrelocc+j, 1:(nrecocc*ndest)])

}

}

### priors

s ~ dunif(0,1)

for(k in 1:ndest){

for(l in 1:nrecocc){

r[k,l] ~ dbeta(ra[k],rb[k])

}

ra[k]~dgamma(0.01, 0.01)

rb[k]~dgamma(0.01, 0.01)

}

}

",fill=TRUE)

sink()

# Define parameters to be monitored

parameters <- c("m","s","r", "ynewlong")

# MCMC settings

niter <- 20000

nthin <- 2

nburn <- 5000

nchains <- 2

initfun <- function(){

ncells <- datax$npop*8*datax$ndest*datax$nrecocc

m0a <- array(runif(ncells, 0,1), dim=c(datax$npop, datax$nbehgroup, datax$ndest, datax$nrecocc))

for(i in 1:datax$npop){

for(j in 1:datax$nbehgroup){

# proportion of birds in A

m0a[i,j,1,1] <- runif(1,0,0.01) # winter

m0a[i,j,1,2] <- runif(1, 0,0.01) # march

m0a[i,j,1,6] <- runif(1, 0, 1) # sept

m0a[i,j,1,7] <- runif(1, 0, 1) # oct

m0a[i,j,1,8] <- runif(1, 0, 0.01) # nov

for(k in 3:datax$ndest){ # in C and D

m0a[i,j,k,5] <- runif(1, 0, 0.01) # juni-aug

}

}

}

list(r=matrix(runif(datax$ndest*datax$nrecocc, 0.001, 0.01), ncol=datax$nrecocc, nrow=datax$ndest),

ra=runif(datax$ndest, 0.2, 2), rb=runif(datax$ndest, 5,10),

s=runif(1, 0.5,1), m0a=m0a)

}

# run jags

mod <- jags(datax, inits=initfun, parameters, "distributionmodelrtime.txt", n.chains = nchains, n.thin = nthin, n.iter = niter, n.burnin = nburn, working.directory=bugsworkingdir)
