# Supplementary material for: A bird distribution model for ring recovery data: where do the European robins go?
Source: Ecol Evol. 2014 Feb 14;4(6):720–31. doi: 10.1002/ece3.977 (PMC3967898; doi:10.1002/ece3.977)
Supplement: Data S2 — The aim of this simulation study was to assess the sample size required to estimate the proportion of birds of the different sets of birds (ij) being in the different regions k during each season q, mijkq. [file ece30004-0720-sd2.pdf]

Data S2: Simulations to assess required sample size

Korner-Nievergelt, F., Liechti, F., Thorup, K. (2014):
A bird distribution model for ring recovery data:
Where do the European robins go?
Ecology and Evolution

February 5, 2014

The aim of this simulation study was to assess the sample size required to estimate the proportion of birds of the different sets of birds (ij) being in the different regions k during each season q, m_{ijkq}. To do so, we simulated data assuming realistic values for monthly survival probability and recovery probability and we varied the number of birds released per month and region.
True monthly survival was set to 0.93 and true recovery probabilities were set to 0.0006 for Fennoscandia, 0.001 for central Europe, 0.0015 for southern Europe, and 0.002 for northern Africa. We assumed constant recovery probability over time. The number of released birds per month was set to 1000, 10000 or 50000, and the number of months without any release was set to 0, 2 or 5. This produced 9 different scenarios which differed in sample size and the distribution of the number of released birds among the months. For each setting, we simulated 20 data sets.

We fitted the model to each simulated set of data using Jags as described in the main text of the manuscript.

For each model fit and for each parameter, we calculated the difference between the estimated and the true parameter value. The mean of these differences over all the simulations and parameters gave the bias and their standard deviation gave the mean squared error (MSE), that is a measurement for precision (Table 1). Because the number of parameters for survival s, recovery probability r_k and the distribution parameter m_{ijkq} differ, the bias and MSE were based on different sample sizes for the different parameters. These sample sizes were 20 for survival, $20*4 = 80$ for recovery probability, and $20 * 2 * 12 * 4 * 8 = 15360$ for m_{ijkq}. In addition, we also give the inclusion probability, which is the proportion of cases where the true value was within the 95% credible interval of the estimate.

Table 1: Average bias (bias.w), mean squared error (mse.w) and inclusion probability (ip.w) for w = the survival parameter s, reencounter probability r and distribution parameter m. n = number or birds released per month and region, n0 = number of monthes without releases, nrec = average total number of recoveries in the simulated data.

n	n0	nrec	bias.s	mse.s	ip.s	bias.r	mse.r	ip.r	bias.m	mse.m	ip.m
50000	0	1487	0.00124	0.00815	0.95	-0.00003	0.00013	0.80	0.00000	0.10325	0.93
10000	0	306	-0.00042	0.01628	0.95	-0.00000	0.00019	0.89	0.00000	0.15173	0.92
1000	0	32	-0.01300	0.03654	0.95	0.00024	0.00065	0.91	0.00000	0.17773	0.92
50000	2	1366	0.00069	0.00957	0.90	-0.00003	0.00011	0.86	0.00000	0.10933	0.93
10000	2	281	-0.00474	0.01272	1.00	0.00000	0.00021	0.92	0.00000	0.15174	0.93
1000	2	25	-0.02323	0.04487	1.00	0.00005	0.00042	0.97	-0.00000	0.17679	0.92
50000	5	1192	0.00064	0.00869	0.95	-0.00003	0.00013	0.86	0.00000	0.11900	0.93
10000	5	234	0.00292	0.02448	0.85	-0.00004	0.00020	0.97	-0.00000	0.15797	0.93
1000	5	23	-0.01255	0.04228	1.00	0.00018	0.00061	0.95	-0.00000	0.17814	0.92

The bias seemed to be close to zero for all parameters. The MSE for survival probability was less than 5% of the true value 0.93 for all scenarios. The MSE decreased with increasing sample size for all model parameters. Inclusion probability seems to be perfectly at 95% for survival probability but

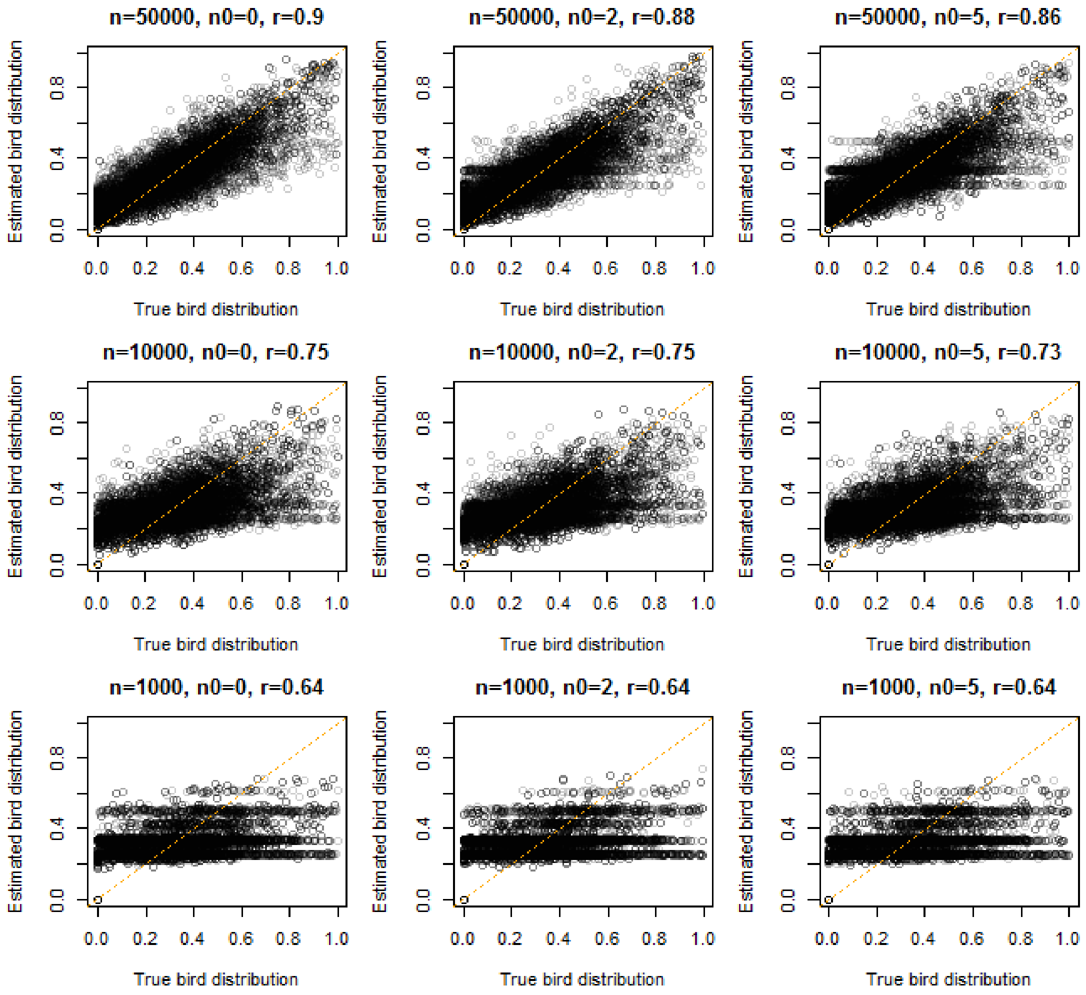

Figure 1: Estimated bird distribution parameters m versus true values for the nine scenarios. In the titles of the panels, n is the number of released virtual birds per month and region, n0 is the number of months without released birds at all, and r is the Pearsons correlation between estimated and true values. The orange broken line is the line y = x.

slightly lower for the other model parameters.

A bias of zero does not mean that the parameter is estimable. When the information in the data is low (due to small sample size) then the estimate equals the prior distribution (which has a bias of zero). Therefore, we plotted the estimated m_{ijkq} against their true values for each scenario separately (Fig. 1).

Regarding sample size, our real data is situated between row one and two and between column one and two. Thus, we can expect that some parameters will be estimable whereas other may not. We therefore, compared the posterior distribution with the prior distribution for each parameter (see main text).

The R- and Bugs-code for the simulation and analyses in this supplementary material can be downloaded from http://www.oikostat.ch/rcode/robin_distributionmodel_supplmat_2_samplesize.r.
