# Supplementary material for: A bird distribution model for ring recovery data: where do the European robins go?
Source: Ecol Evol. 2014 Feb 14;4(6):720–31. doi: 10.1002/ece3.977 (PMC3967898; doi:10.1002/ece3.977)
Supplement: Supplementary file 3 — Data S3. This document contains two figures that show the 95% credible intervals of all proportions presented in the barplot of Fig. 2 of the main text. Fig. S3. (A) Estimated proportions of birds ringed in Fennoscandia during different months (indicated on the left outer axis) being in the four different regions (indicated on the bottom) during 8 different seasons (x-axis). (B) Legend as in Fig. S3a but for birds released in central Europe. [file ece30004-0720-sd3.docx]

**Data S3: Credible intervals of the distribution parameters**

Korner-Nievergelt, Liechti, Thorup (2014): A bird distribution model for ring recovery data: Where do the European robins go? Ecology & Evolution

This document contains two figures that show the 95% credible intervals of all proportions presented in the barplot of Fig. 2 of the main text.


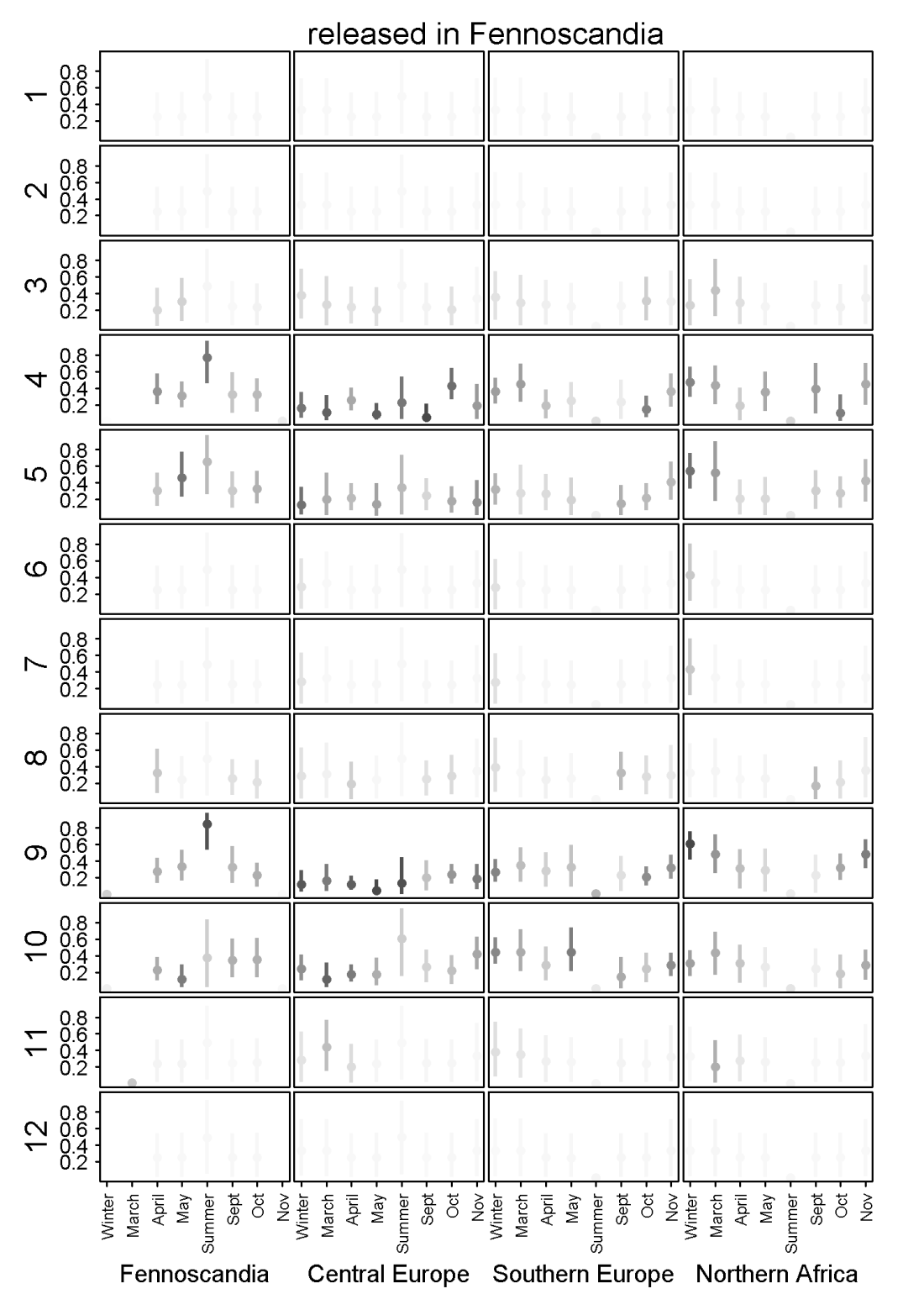


Fig. S3a. Estimated proportions of birds ringed in Fennoscandia during different months (indicated on the left outer axis) being in the four different regions (indicated on the bottom) during 8 different seasons (x-axis). The vertical bars give the 95% CrI. Grey scales proportional to the overlap between prior and posterior distribution. White (invisible) means that the posterior equals the prior (no information in the data). Darker gray corresponds to more data-informed estimates.


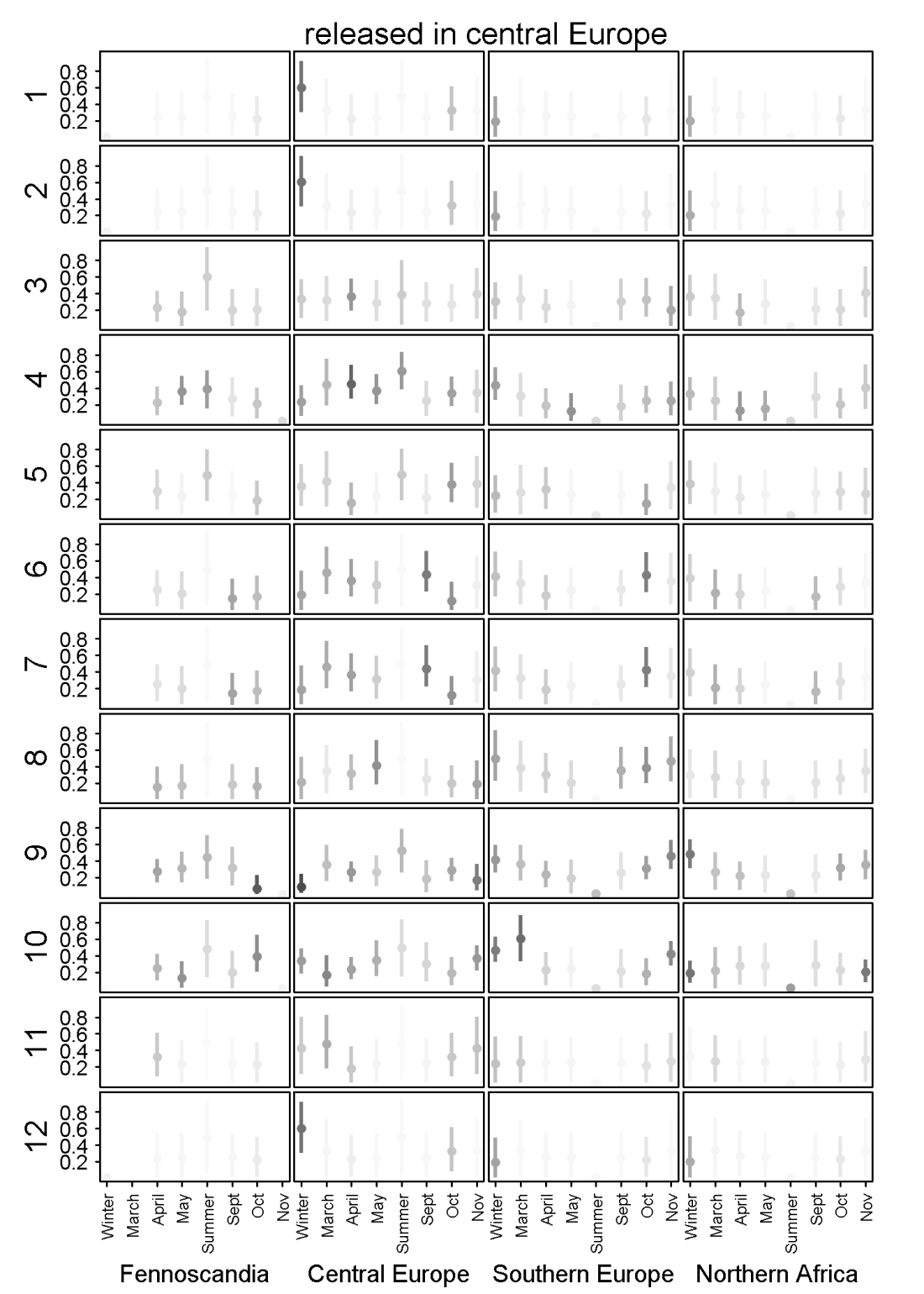


Fig. S3b. Legend as in Fig. S3a but for birds released in central Europe.
