# Supplementary material for: A bird distribution model for ring recovery data: where do the European robins go?
Source: Ecol Evol. 2014 Feb 14;4(6):720–31. doi: 10.1002/ece3.977 (PMC3967898; doi:10.1002/ece3.977)
Supplement: Supplementary file 4 — Data S4. Diagnostic figures from posterior predictive model checking for two models: (1) model assuming constant recovery probability over time (not presented in the main text), (2) model assuming season-dependent recovery probability (as presented in the main text). Fig. S1. Mean and 95% interval of the posterior predictive distributions of the number of recoveries [log ()] centered around the observed number of recoveries in each region for each season (bottom axis) for birds ringed in Fennoscandia during a specific month (left outer axis). Fig. S2. Legend as in Fig. S1 but for birds ringed in central Europe. [file ece30004-0720-sd4.docx]

**Data S4: Posterior predictive model check**

Korner-Nievergelt, Liechti, Thorup (2014): A bird distribution model for ring recovery data: Where do the European robins go? Ecology & Evolution

Diagnostic figures from posterior predictive model checking for two models: 1) model assuming constant recovery probability over time (not presented in the main text), 2) model assuming season-dependent recovery probability (as presented in the main text). At the end, we shortly discuss why we favored the model with season-dependent recovery probabilities.


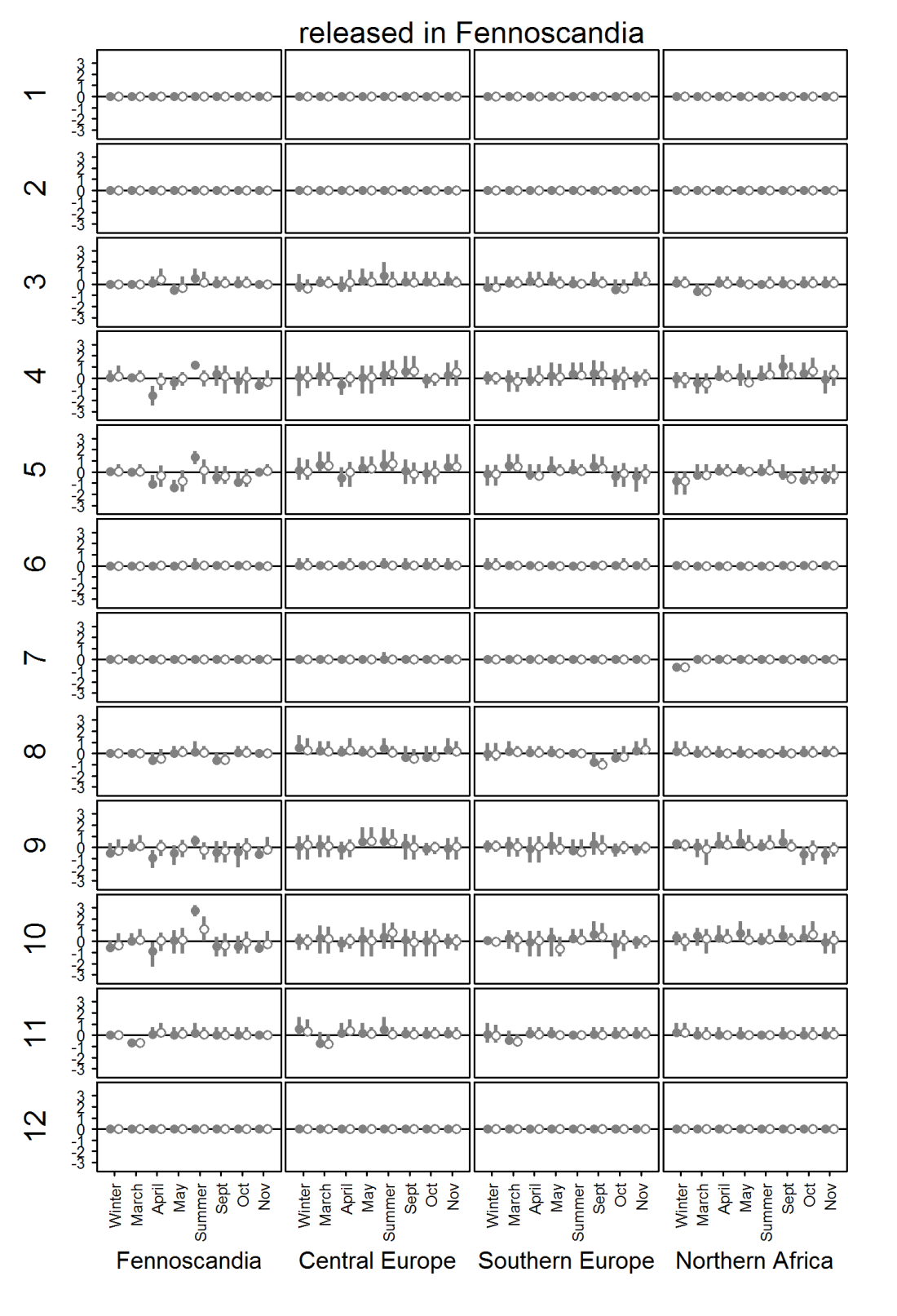


Fig. S1. Mean and 95% interval of the posterior predictive distributions of the number of recoveries (log(*R^new^_ijkq_*+1)) centred around the observed number of recoveries in each region for each season (bottom axis) for birds ringed in Fennoscandia during a specific month (left outer axis). Filled grey = model with constant recovery probability, open grey = model with season-dependent recovery probability.


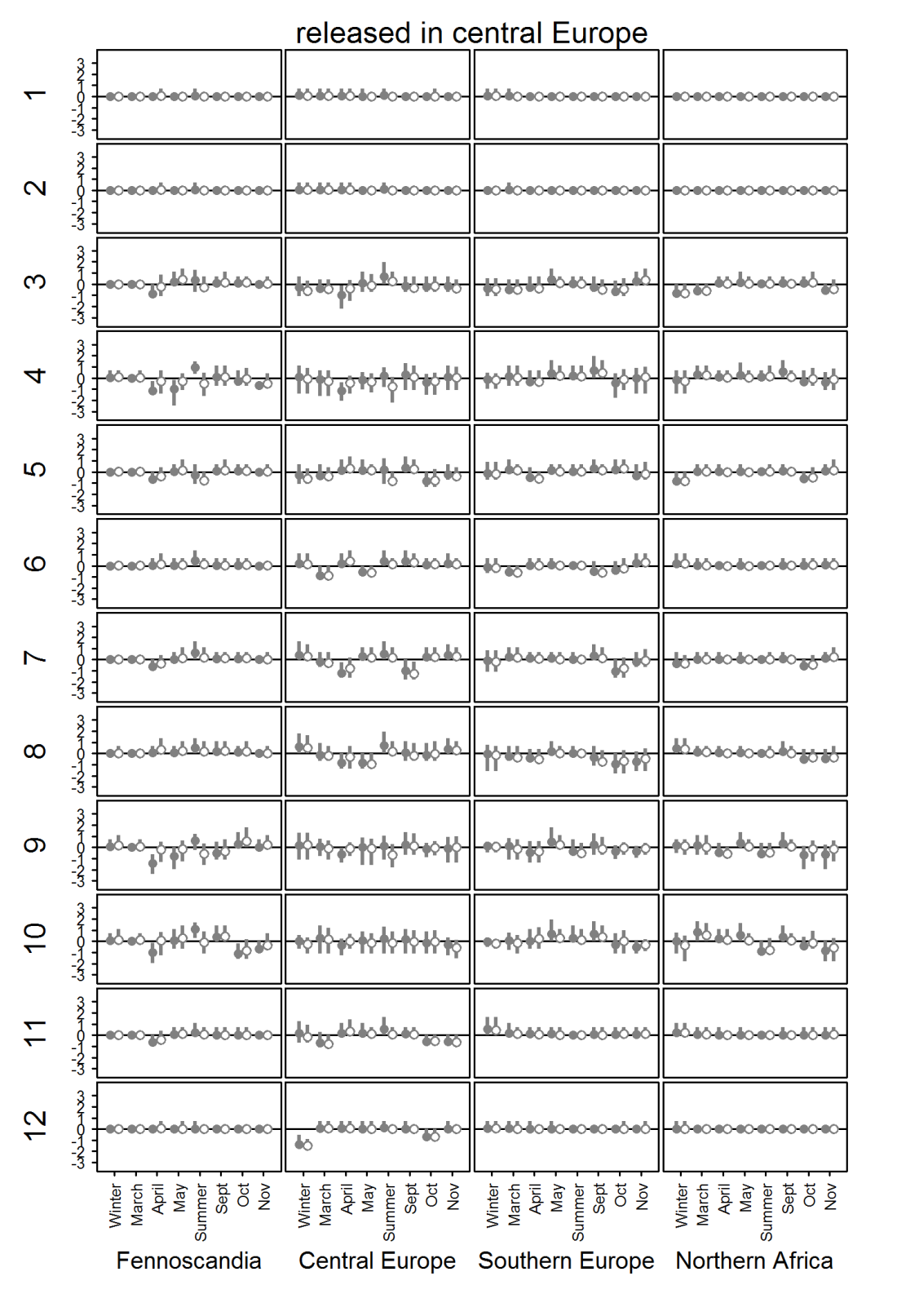


Fig. S2. Legend as in Fig. S1 but for birds ringed in central Europe.

The two models we fitted differed in the number of parameters used to describe recovery probability. The season-constant recovery probability model included four different parameters for recovery probability, one for each region. In this model we assumed that recovery probability was constant during the year. The season-dependent recovery probability model included 24 parameters for recovery probability, for each of the four regions 8 different probabilities for each season. We clearly favor the season-dependent recovery probability model, since recovery circumstances changes along the course of a year: during migration robins may be more prone to collide with human made structures than during the breeding season (e.g. Liechti, Guélat & Komenda-Zehnder 2013), or hunting pressure is clearly enhanced during hunting seasons. Also the DIC favors the season-dependent recovery probability model (∆DIC = 384, Spiegelhalter *et al.* 2002).

Liechti, F., J. Guélat, and S. Komenda-Zehnder. 2013. Modelling the spatial concentrations of bird migration to assess conflicts with wind turbines. Biological Conservation 162:24-32.

Spiegelhalter, D. J., N. G. Best, B. P. Carlin, and A. van der Linde. 2002. Bayesian measures of model complexity and fit. Journal of the Royal Statistical Society, Series B 64:1-34.
