# Supplementary material for: A bird distribution model for ring recovery data: where do the European robins go?
Source: Ecol Evol. 2014 Feb 14;4(6):720–31. doi: 10.1002/ece3.977 (PMC3967898; doi:10.1002/ece3.977)
Supplement: Data S5 — The aim of this simulation study was to assess the influence of nonconstant survival on the estimates of the model parameter, as our model assumes constant survival probability. [file ece30004-0720-sd5.pdf]

Data S5: Simulations to assess effect of nonconstant survival

Korner-Nievergelt, F., Liechti, F., Thorup, K. (2014):
A bird distribution model for ring recovery data:
Where do the European robins go?
Ecology and Evolution

February 5, 2014

The aim of this simulation study was to assess the influence of non-constant survival on the estimates of the model parameter, since our model assumes constant survival probability. We simulated data assuming a monthly survival probability s of 0.98 for the winter and summer months (November to February, May to August) and of 0.85 for the months of migration. These values were taken from the study on *Dendroica caerulescens* by Sillett and Holmes (2002). The true recovery probabilities were set to 0.001, 0.004, 0.003 and 0.001 for Scandinavia, central Europe, southern Europe and northern Africa respectively. Recovery probability was held constant over time in the data simulation but assumed to vary between the seasons in the model. The true values for the parameters m_{ijkq} were drawn at random from their prior distributions. The number of released birds per month was set to 100000. We simulated 20 data sets.

We fitted the model to each simulated set of data using Jags as described in the main text of the manuscript.

For each model fit and for each parameter, we calculated the difference between the estimated and the true parameter value. The mean of these differences over all the simulations and parameters gave the bias and their standard deviation gave the mean squared error (MSE), that is a measurement for precision (Table 1). Because the number of parameters for survival s, recovery probability r_k and the distribution parameter m_{ijkq} differ, the bias and MSE were based on different sample sizes for the different parameters. These sample sizes were 20 for survival, $20 * 4 * 8 = 640$ for recovery probability, and $20*2*12*4*8 = 15360$ for m_{ijkq}. In addition, we also give the inclusion probability, which is the proportion of cases where the true value was within the 95% credible interval of the estimate. We compared the estimated monthly survival probability $\hat{s}$ (which was assumed to be constant by the model) with the average of the different 'true' monthly survival probabilities which was 0.94.

We fitted the model ignoring sex to each simulated set of data using Jags as described in the main text of the manuscript.

Table 1: Average bias (bias.w), mean squared error (mse.w) and inclusion probability (ip.w) for w = the survival parameter s, reencounter probability r and distribution parameter m. n = number or birds released per month and region, nrec = average total number of recoveries in the simulated data.

n	nrec	bias.s	mse.s	ip.s	bias.r	mse.r	ip.r	bias.m	mse.m	ip.m
100000	5041	0.00406	0.00403	0.90	0.00062	0.00265	0.00	0.00000	0.08681	0.95

The estimated distribution parameters m_{ijkq} seem to be unbiased and their credible intervals correctly estimated (inclusion probability is close to 0.95). However, the inclusion probability for the estimated survival was smaller than when data is simulated under the assumption of constant survival

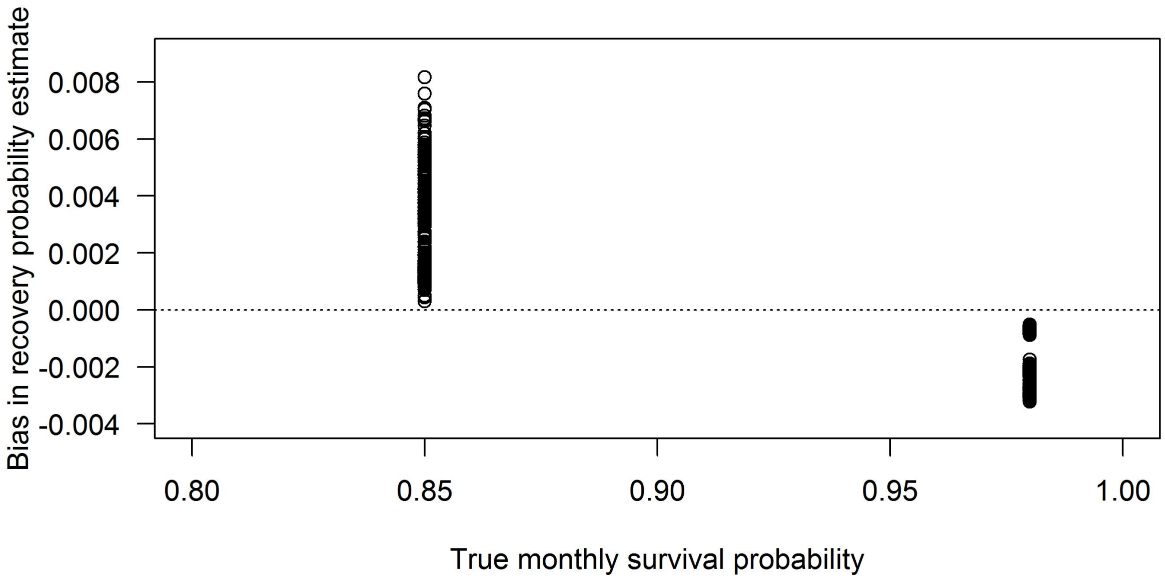

Figure 1: Bias in the estimate for recovery probability ($\hat{r} - r$) versus the underlying monthly survival probability used to simulate the data.

probability (as e.g. in supplementary material 2) and almost no credible interval of the estimate of recovery probability included the true parameter value (Table 1).

Heterogeneity in survival probability that was not accounted for in the model resulted in biased estimates for season-specific recovery probabilities (Fig. 1). For seasons with low survival, recovery probability was overestimated and for seasons with high survival it was underestimated. The estimated bird distribution parameters were not affected by heterogeneity in survival probability.

References

Sillett, T. S., and R. T. Holmes. 2002. Variation in survivorship of a migratory songbird throughout its annual cycle. Journal of Animal Ecology **71**:296–308.
