# Supplementary material for: A bird distribution model for ring recovery data: where do the European robins go?
Source: Ecol Evol. 2014 Feb 14;4(6):720–31. doi: 10.1002/ece3.977 (PMC3967898; doi:10.1002/ece3.977)
Supplement: Data S6 — The aim of this supplementary material is to assess the effect of sex-specific migration on the model estimates when sex is not identified. [file ece30004-0720-sd6.pdf]

Data S6: Simulations to assess effect of differential migration

Korner-Nievergelt, F., Liechti, F., Thorup, K. (2014):
A bird distribution model for ring recovery data:
Where do the European robins go?
Ecology and Evolution

February 5, 2014

The aim of this supplementary material is to assess the effect of sex-specific migration on the model estimates when sex is not identified. To do so, we first show analytically that differential migration within the sets of birds released at the same region i during the same month j does not produce bias in the parameter estimates for a very simple case with only two sets of birds, two recovery regions and one recovery occasion. Then, we used simulated data to show that this also is true for more complex data as our example data with 24 sets of birds, 4 recovery regions and 8 recovery occasions. We simulated data for males and females separately assuming that females migrate farther south than males and fitted our model to the data ignoring sex.

1 Analytic assessment

Let $n♂_A$, $n♀_A$, $n♂_B$, and $n♀_B$ be the number of released males and females in the regions A and B. Let's further assume that $m♂_{ik}$ and $m♀_{ik}$ are the proportions of birds migrating from region i to region k for the two sexes, with $i = (A, B)$ and $k = (C, D)$, and r_C and r_D are the recovery probabilities in C and D. The recovery probabilities r_C and r_D and the distribution parameters $m♂_{ik}$ and $m♀_{ik}$ are unknown.

The number of males from A migrating to C is $m♂_{AC}{*}n♂_A$, and $m♂_{AD}{*}n♂_A$ males from A migrate to D. Similarly, the numbers of females migrating from A to C is $m♀_{AC} * n♀_A$.

If sex cannot be identified, the number of recovered birds from i in k is

$$R_{ik} = (m♂_{ik} * n♂_i + m♀_{ik} * n♀_i) * r_k \quad (1)$$

Thus, the total number of birds migrating from i to k is

$$R_{ik}/r_k = (m♂_{ik} * n♂_i + m♀_{ik} * n♀_i) \quad (2)$$

Because we assume that no bird has gone somewhere else than to C and D, the sum of the birds (males and females) that have migrated from i to C and D equals the total number of birds released in i.

$$n♂_i + n♀_i = (m♂_{iC} * n♂_i + m♀_{iC} * n♀_i) + (m♂_{iD} * n♂_i + m♀_{iD} * n♀_i) \quad (3)$$

The total number of birds released in i is $n♂_i + n♀_i = n_i$. We insert equation (2) in (3) to obtain the equation system:

$$\begin{aligned} n_A &= R_{AC}/r_C + R_{AD}/r_D \quad (4) \\ n_B &= R_{BC}/r_C + R_{BD}/r_D \quad (5) \end{aligned}$$

This system of two linear equations contains two unknowns (r_C and r_D). Such a system has exactly one solution for $1/r_C$ and $1/r_D$ if the determinant of the coefficients of the linear system is different from zero:

$$\begin{vmatrix} R_{AC} & R_{AD} \\ R_{BC} & R_{BD} \end{vmatrix} \neq 0$$

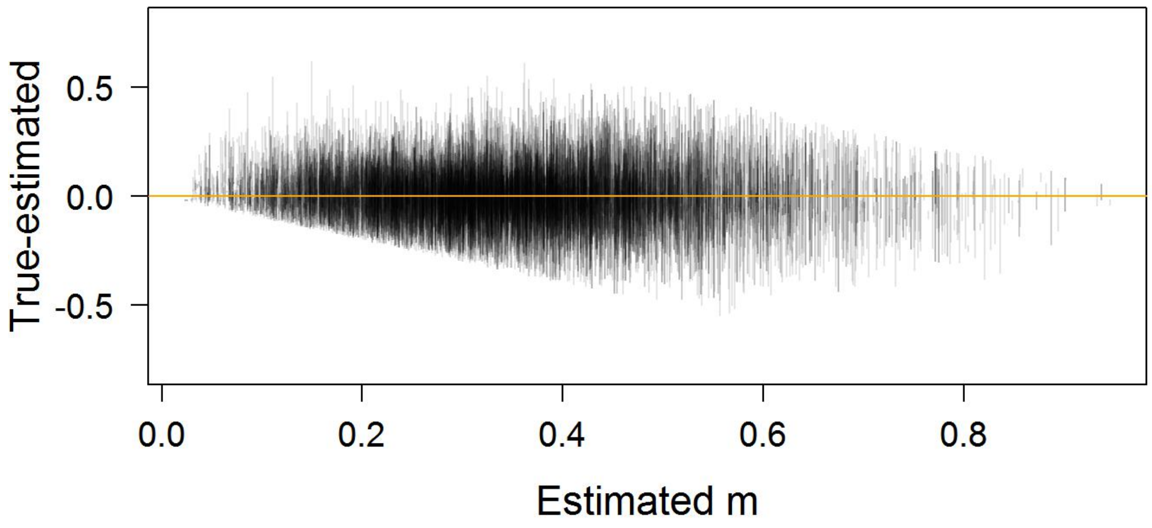

Figure 1: Estimated distribution parameters m (= proportions of birds being in the different regions) in relation to true values for males and females for the models fitted to simulated data with different distribution parameters between the sexes. The vertical bars connect the true values for males and females centered around the estimate (orange line).

The determinant deviates from zero as soon as the proportions of birds from A and B migrating to the regions C and D differ somewhat (Korner-Nievergelt et al., 2010). Therefore, the recovery probabilities r_C and r_D are estimable in most real data (when the sets of birds released are chosen appropriately). We, then, can insert the estimated r_k into equation (2) to get an estimate for the total number of birds (males and females) migrating to k, which equals $(m♂_{ik} * n♂_i + m♀_{ik} * n♀_i)$. Thus, r_k is identifiable and the model can estimate $m_{ik} = (m♂_{ik} * n♂_i + m♀_{ik} * n♀_i)/n_i$, which is the proportion of birds from i in k independent of sex. In the case of a sex-ratio of 1:1, i.e. $n♂_i = n♀_i = n_i/2$, m_{ik} becomes $(m♂_{ik} * n_i/2 + m♀_{ik} * n_i/2)/n_i$ =m$♂_{ik}/2 + m♀_{ik}/2$ the arithmetic mean between the sex-specific distribution parameters. In the case of non-equal sex-ratio, the estimate is a weighted mean.

To conclude, if a species shows sexual different migration but the sexes cannot be identified, the simplified model is able to estimate the recovery probabilities in the different regions without bias. As a consequence, also the overall proportions of birds migrating to the different regions is estimable without bias.

2 Simulation

To simulate the data we assumed realistic values for monthly survival probability and recovery probability and we used four different sample sizes.

True monthly survival was set to 0.93 and true recovery probabilities were set to 6×10^{-4} for Fennoscandia, 0.001 for central Europe, 0.0015 for southern Europe, and 0.002 for northern Africa. We assumed constant recovery probabilities over time. We assumed no differences in survival or recovery probability between the sexes. The true values for the parameters m_{ijkq} were drawn at random from their prior distributions. Separate values for males and females were drawn. Because the true m_{ijkq} were drawn as random values from distributions (instead of fixing each of these

parameters to a predefined value), the differences in the true m-values between the sexes varied between the data sets (the differences between the sexes for each m-value are visualized in Figure 1).

The number of released birds per month was set to 10^5. We assumed a sex-ratio of 1:1. We simulated 20 data sets.

In order to see the effect of differential migration on our model, we repeated the simulation with the same number of birds released, the same true values for survival and recovery probability but with no difference in the migration pattern between the sexes.

We fitted the model ignoring sex to each simulated set of data using Jags as described in the main text of the manuscript.

For each model fit and for each parameter, we calculated the differences between the estimated and the true parameter values. The mean of these errors over all the simulations and parameters gave the bias and their standard deviation gave the mean squared error (MSE), that is a measurement for precision (Table 1). Because the number of parameters for survival s, recovery probability r_k and the distribution parameter m_{ijkq} differ, the bias and MSE were based on different sample sizes for the different parameters. These sample sizes were 20 for survival, $20*4 = 80$ for recovery probability, and $20*2*12*4*8 = 15360$ for m_{ijkq}. In addition, we also give the inclusion probability, which is the proportion of cases where the true value was within the 95% credible interval of the estimate.

Table 1: Average bias (bias.w), mean squared error (mse.w) and inclusion probability (ip.w) for w = the survival parameter s, reencounter probability r and distribution parameter m. The number of released birds per month and region was 100000 in both data sets. diffmig = indicates whether differential migration was present in the data, nrec = average total number of recoveries in the simulated data.

diffmig	nrec	bias.s	mse.s	ip.s	bias.r	mse.r	ip.r	bias.m	mse.m	ip.m
no	5981	-0.00132	0.00426	1.00	-0.00001	0.00007	0.90	-0.00273	0.07215	0.94
yes	6020	-0.00037	0.00410	0.95	-0.00001	0.00007	0.94	-0.00277	0.06983	0.96

The average bias seems to be close to zero for all model parameters. The 95% credible intervals include the true values with a probability of closely 0.95 for all parameters (Table 1). The estimate for the distribution parameter m had a slight negative average bias (when assuming that the true value for m is the arithmetic mean between the male and female parameter value, which may be an explanation for the bias). Concerning the bias and precision of the parameter estimates, there was no difference discernible between the models fitted to data including differential migration and those models fitted to homogeneous data sets.

The estimated bird distribution parameters seem to lie on average between the true values for males and females (Fig. 1).

To conclude, estimated model parameters seem to be quite robust when not accounting for differential migration in the model.

References

Korner-Nievergelt, F., M. Schaub, K. Thorup, M. Vock, and W. Kania. 2010. Estimation of bird distribution based on ring re-encounters: precision and bias of the division coefficient and its relation to multi-state models. Bird Study, **57**:56–68.
